# Silver Ions in the Treatment of Local Infections

**DOI:** 10.1155/MBD.1999.311

**Published:** 1999

**Authors:** Robert O. Becker

**Affiliations:** Department of Orthopaedic Surgery State University of New York Health Science Center at Syracuse 550 Harrison Street Syracuse New York 13202 USA

## Abstract

A study of the use of free silver ions as an antibacterial and antifungal agent administered to infected local wounds has been conducted over the past two decades. A variety of iontophoretic techniques has been employed utilizing either pure silver wires or several types of silvered nylon fabrics as anodes in a direct current electrical circuit. A new type of silver nylon has recently been evaluated for the same use without iontophoretic current. *In vitro* studies of both techniques have demonstrated an effective, broad spectrum antibiotic effect including most silver-resistant strains. Over 100 cases of recalcitrant osteomyelitis have been treated with an overall success rate of approximately 65% and no evidence of argyria.


### SILVER IONS IN THE TREATMENT OF LOCAL INFECTIONS

Robert O. Becker

Department of Orthopaedic Surgery, State University of New York,
Health Science Center at Syracuse, 550 Harrison Street, Syracuse, New York 13202, USA

### ABSTRACT

A study of the use of free silver ions as an antibacterial and antifungal agent administered to infected local wounds has been conducted over the past two decades. A variety of iontophoretic techniques has been employed utilizing either pure silver wires or several types of silvered nylon fabrics as anodes in a direct current electrical circuit. A new type of silver nylon has recently been evaluated for the same use without iontophoretic current. *In vitro* studies of both techniques have demonstrated an effective, broad spectrum antibiotic effect including most silver-resistant strains. Over 100 cases of recalcitrant osteomyelitis have been treated with an overall success rate of approximately 65% and no evidence of argyria.

### INTRODUCTION

Silver has long been known to have anti-infective properties. Modern usage began with Crede's technique of dilute silver nitrate as prophylasis of gonorrhea opthalmicum in 1881[1]. The first recoded use of silver in surgery was Halsted's classic paper in 1913, [2] in which he reported the use of silver foil as the initial dressing for fresh surgical incisions. He demonstrated its anti-bacterial effects and stated, " we are wedded to its use, and I know of nothing which could quite take its place,". In the same paper, Halsted first advocated the use of rubber gloves and rubber tube surgical drains both of which were immediately accepted by the surgical community and are in use today. For unknown reasons the use of silver foil never quite became popular. In 1967 Fox [3] reported on the use of a silver-sulfadiazine compound in an ointment for the treatment of infected burns which remains the major agent for this use today.

The introduction of natural and synthetic antibiotics totally changed the treatment of systemic diseases such as pneumonia and septicemia. However,their direct use in localized, infected wounds proved unsatisfactory due to their irritative and allergic effects on tissues. As a result, such infections were treated with systemic administration of antibiotics. While this involved the infusion of these agents throughout the body to treat a strictly local condition, it was initially effective. Now, however, the nature of infectious processes has been greatly changed with the introduction of mutated forms resistant to the action of most antibiotics. As a result the effective treatment of local infections has become much more difficult requiring systemic administration of large doses of multiple antibiotics often with attendant undesirable side effects.

Even after the introduction of antibiotics, infections of bone ( osteomyelitis ) remained difficult to treat because the limited blood supply to this tissue precluded obtaining adequate bone levels of systemically administered antibiotics. In the early 1970's this situation became more complex with the appearance of mixed infections including resistant bacteria in such wounds. Today, the only effective treatment remains primarily surgical with adequate wound debridement, leaving the wound open and supplementing the treatment with appropriate systemic antibiotics. A method to introduce a competant, broad spectrum, antibacterial agent directly into the infected bone was obviously desirable and I considered some type of iontophoresis.

### METHODS

In earlier studies on electrical stimulation of bone growth I had noted that in a circuit generating current through a body part with metallic electrodes, the anodal current consisted of free metallic ions from the anode. These ions, driven along the voltage gradient, penetrated one to two cm into the conducting medium before other conducting mechanisms took over. This appeared [illegible]o be a possible technique for introducing free silver ions from a metallic silver anode into an oste[illegible]yelitic lesion. Starting in 1974, *in vitro* experimentation, indicated that the technique was indee[illegible]iable, demonstrating an effective, broad spectrum, antibacterial and antifungal effect with no [illegible]vious adverse effect on living mammalian cells. [4-7] Animal experimentation also indicated a useful effect on experimental osteomyelitis with no evident harmful side effects.[8] Clinical use was begun in 1977 and the technique proved to be highly effective and not productive of adverse effects.[9-11] Clinical experimentation has continued to today and devices and techniques of administration have continued to be refined.

Initially, 99.99% silver wire anodes were used in the treatment of small, localized, infected bone lesions with the wire surgically inserted into the lesion and the administration of 0.1 volts between it and a remote surface cathode. While this was quite successful, it was not applicable to the majority of osteomyelitic lesions with open wounds ranging in size from 4 to 20 square inches with infected bone visible in the wound. A flexible, nylon fabric material infiltrated with silver produced by a chemical deposition process was located and proved useful in such extensive lesions. This material was a commercial product manufactured by Swift Textile Metalizing Co. Bloomfield, CT for use as a flexible connector in the elecronics industry. Consequently, the silver deposit contained a variety of other trace metals, which however, did not appear to be a problem in its use. The silver fabric was sinply cut generously to fill the wound cavity, wet with sterile water, packed into the wound with a "tail" that exited the wound and was connected to a battery operated, constant voltage generator limited to 0,9 V. Electrodes were changed daily and treatment was continuous except for daily electrode changes. The silver nylon was non-adherant to the wound, dressing changes were painless and the material left no residue in the wound. If, prior to iontophoresis therapy, the wound required surgical debridement the patients were placed on appropriate systemic antibiotics for the initial 3 post operative days to prevent circulatory bacterial spread. Thereafter, antibiotics were found to be not required. Daily bacterial cultures were taken from the wounds and indicated control of the infection by the 7th to 10th day of treatment. Often the 10 day culture indicated a sterile wound, despite no sterile precautions being used during wound care.

It was noted that for wound areas smaller than 2-4 square inches the voltage level of 0,9V was productive of local electrolysis and while antibiotic effects were adequate, wound healing was impeded. Studies indicated the requirement for the applied voltage to be linearly scaled to the wound size, with 0.1 V for wounds 2 square inches and lower in size to a maximum of 0.9 V for wounds of 10 square inches and larger. This completely avoided the interference with wound healing. The fabric tail connection between the moistened anode and the electrical generator must be kept dry to prevent conduction through the aqueous medium. This is accomplished by cutting the "tail" of silver fabric connecting the dressing to the generator adequate in length to protrude through the dressing to a dry area. It was also noted that this tail must be of at least one inch in width initially and tapered wider as it enters the anode to avoid an abrupt step in in the current density distribution.

Five years ago a new silvered nylon material, maufactured by Omnishield Co., Clark Summit, PA became available which contained no trace elements by X-ray spectroscopy. Additionally, a proprietory process for silvering the fabric is employed that results in the deposition of nano -crystals of silver rather than a metallic coating.[Fig.1] This deposit has proven to be capable of spontaneously releasing large numbers of free silver ions into the wound environment without the application of electrical current. This material has been found to be as clinically effective as the iontophoretic technique with equivalent antibiotic effects. The technique of use is similar except no connection to an external elecrical source is required. At present, this silver nylon is simply cut generously to fit into the wound, wet with sterile water, placed securely in the wound and covered with a standard surgical dressing ( also moistened ), and afixed in place with an external dressing. It is important to mantain a level of moisture at the actual wound level as the silver deposit stops emitting ions if it becomes dry. Initially, the dressings are changed twice daily when treatment is first started. After control of infection is obtained, once per day is generally sufficient. Hospitalization has not been necessary. Patients are usually capable of changing their own dressings or a relative or local care giver can do so with minimal instruction.

### RESULTS

Over 100 cases of osteomyelitis, many with non-unions of bone in the wound, were treated with the iontophoretic technique with an approximately 75% success rate in controlling the infection permitting the wounds to heal without drainage. Healing of most non-united fractures present in the wound was also noted. The majority of failures to completely control infection were due to retained fragments of dead bone in the deeper portion of the wound. No undesirable side effects were noted and there was no evidence for local or systemic argyria.

The use of the new type of silver nylon without electrical current has thus far been limited to a smaller number of infected traumatic or diabetic wounds smaller than 4 sq. in. that had failed to respond to systemic antibiotics. This use has been found to be very effective in treating all traumatic wounds contaminated with resistant bacteria without the need for concomitant intravenous antibiotics. 60% of similarly infected diabetic wounds have been successfully treated. However, it must be noted that there are certain types of diabetic ulcers and spontaneous tissue necrosis that are not primarily infected with micro-organisms. These appear to be primarily disturbances in the healing control systems and the silver materials have little effect on these conditions. While some instances of silver resistant bacteria, particularly E. coli and Enterobacter, have been reported these have not been encountered in my series thus far.

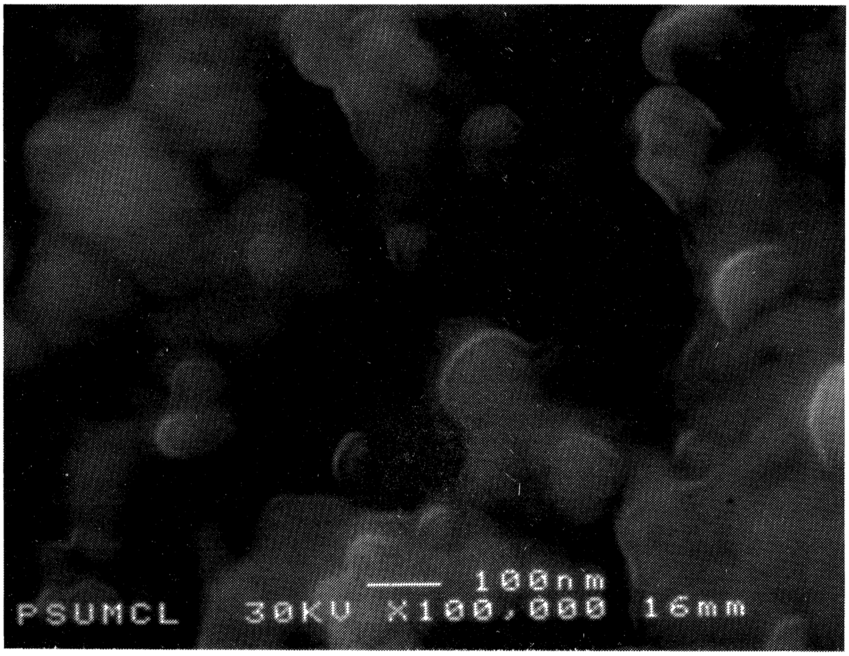

Figure 1. Scanning electron micrograph of the silver deposit on a single nylon fiber. The silver deposit is composed of small units averaging 100 nm which appear to be made of a number of single crystals. Crystal angles can be seen on the edges of individual deposits. Magnification indicated by the scale bar.

### DISCUSSION

Both the silver iontophoresis technique and the simple application of the silver fabric in wound care utilize no agent other than free silver ions given off into the wound as the primary anti-bacterial agent. The method appears to have several advantages not shared by other agents. At present, silver ions appear to have the broadest spectrum of anti-bacterial activity The level of free silver ions produced in the wound is greater than with any silver compound presently available. The method of use is simple and dressing changes are easy and non-painful. The silver nylon material is inexpensive and while commercial versions will obviously be more complex, costs should be very competative.

There are two potential problems with the use of any silver antibiotic usage. First, it does appear that silver resistance can occur in at least some bacterial strains. I believe it is extremely important to avoid this circumstance since it appears that, at least in the near future, silver ions may soon be the only remaining effective agent for clinical use. There appears to be a tendency to develop other silver compounds that produce low levels of silver ions in the wound environment or have characteristics permitting long term application with fewer dressing changes as a cost saving measure. Both of these characteristics would appear to favor the development of additional silver resistant strains.

Some of this effort may be directed to avoiding the development of argyria. While much of the pathophysiology of argyria remains obscure, the primary pathology is due to the deposition of metallic silver in the skin, possibly under the influence of light converting a silver compound into metallic silver. It would appear obvious that since silver ions are a highly reactive agent, they cannot circulate in the blood stream unless they are present as a compound that dissociates under the effect of light. The silver nylon application, either iontophoretic or as simple dressings releases only free silver ions, in relatively large amounts directly into the wound tissues where, it is believed, the majority combine with any available chemical agent located therein ( including bacteria ) and remain "locked" *in situ* . In a very few cases, I had occasion to re-enter a previously iontophoretically treated wound. Random soft tissue biopsies from the site demonstrated a few metallic deposits encased in fibrous capsules. No patient treated over the past 20 years with either technique has demonstrated clinical, dermal argyria.

### CONCLUSION

The silver nylon antibacterial therapy appears to offer some important advantages. The free silver ions demonstrate a very wide spectrum of activity. The high concentration of such ions produced in the wound would seem to mitigate against the development of resistant strains of bacteria while at the same time the high level of activity of the silver ions appears to contain them within the wound area obviating the development oif argyria. The ease of usage and the painless nature of the dressing changes make the technique very acceptable to the patient. As a commercial product in optimal form the therapy should be economical.

### ACKNOWLEDGEMENT

The author thanks Mr. John Scofield of OMNISHIELD for making available the scanning electronmicrograph.

### REFERENCES

1. Crede, C.S.F. *Arch. Gynackol.* **17**; 50, 1881

2 Halsted, W.S. *J. Am. Med Assoc* . **60**, 1119-1126, 1913

3. Fox, C.L. Jr. *Mod. Treatment* **4**; 1259-1261, 1967

4 Barrenco, S.D.; Spadaro, J.A,; Berger, T.J.; Becker, R.O. *Clin. OrthO. Rel. Res.* **100**; 250-255,1974

5. Spadaro, J.S.; Berger, T.J. Barrenco, S.E.; Chapin,S.E.; Becker, R.O. *Antimico. Agen. Chemo.* **6**; 637-642, 1974.

6. Berger, T.J.; Spadaro, J.A. Chapin, S E.; Becker, R.O. *Antimicro. Agen. Chemo.* **9;** 357-358, 1976.

7. Berger,T,J,; Spadaro, J.A.; Bierman, R.; Chapin, S.E.; Becker, R.O. *Antimicro. Agen. Chemo.*; **10**, 856-860, 1976.

8. Spadaro, J.A.; Becker, R.O. *Trans. 22d Ann Meet. Ortho. Res. Soc.* 114, 1976

9. Becker, R.O.; Spadaro, J.A. *J. Bone Joint Surg.* **60A**; 871-881, 1981.

10. Webster, D.A.; Spadaro, J.A.; Becker, R.O.; Kramer, S. *Clin. Ortho. Rel. Res.* **161**; 105-114, 1981.

11. Becker, R.O. in Book *Surgery of the Musculoskeletal System*, Editor, Evarts, C.M.C., Churchill Livingstone, New York, 197-208, 1983.

**Received: October 1, 1998 -**
**Accepted in final form: November 29, 1998**

